# Enhanced excitonic emission efficiency in porous GaN

**DOI:** 10.1038/s41598-018-34185-1

**Published:** 2018-10-25

**Authors:** Thi Huong Ngo, Bernard Gil, Tatiana V. Shubina, Benjamin Damilano, Stéphane Vezian, Pierre Valvin, Jean Massies

**Affiliations:** 1 Laboratoire Charles Coulomb, CNRS and Université de Montpellier, CC074, 34095 Montpellier, Cedex 5 France; 2 Faculty of Science and Technology, Meijo University, 1-501 Shiogamaguchi, Tenpaku-ku, Nagoya 468-8502 Japan; 3 Ioffe Institute, 194021 St Petersburg, Russia; 4 Université Cote d’Azur, Centre de Recherche sur l’Hetero-Epitaxie et ses Applications– CNRS– Rue Bernard Gregory, Sophia, Antipolis, 06560 Valbonne France

## Abstract

We investigate the optical properties of porous GaN films of different porosities, focusing on the behaviors of the excitonic features in time-integrated and time-resolved photoluminescence. A substantial enhancement of both excitonic emission intensity and recombination rate, along with insignificant intensity weakening under temperature rise, is observed in the porous GaN films. These observations are in line with (**i**) the local concentration of electric field at GaN nanoparticles and pores due to the depolarization effect, (**ii**) the efficient light extraction from the nanoparticles. Besides, the porosification enlarges the surface of the air/semiconductor interface, which further promotes the extraction efficiency and suppresses non-radiative recombination channels. Our findings open a way to increasing the emission efficiency of nanophotonic devices based on porous GaN.

## Introduction

The extraction efficiency of light emitting devices is limited by various photonic effects that operate (*i*) at the interfaces between different materials assembled to realize the devices and (*ii*) at the interface between the air and a cap layer. A lot of different approaches including the use of shaped substrates or the texturing of the surface of the commercial devices have been used to compete against light refraction effects and to enhance the extraction efficiency. Advantage was also taken of photonics to adapt the design of the device so that both the heart of the light emitter and the air-material interface are located at antinodes of the electromagnetic field^1^.

Porous semiconductors are known to be excellent light-emitters; even the indirect band-gap silicon, that when made porous emits a strong reddish photoluminescence (PL)^2^. This emission is not dominantly related with the confinement effects, but it is obviously correlated with surface contamination effects by oxygen or OH radicals^2^. As a cheap source for light emission, porous Si is promising for photonic applications. By tuning the porosity, in other words, by changing the silicon to air proportions, the dielectric constant of porous Si can be so precisely varied and controlled that highly reflecting Bragg mirrors and microcavities with very high finesses can be very cheaply realized^3^. This paves the way for experimental investigations in the area of fundamental optics^4^, or for realizing the low-cost advanced biosensors for detection of macromolecules in general^5^, or, more specifically, for using in the area of diabetology for the calibration of glucose oxidase^6^. Regarding wide band gap semiconductors, very impressive results were recently published concerning white light emission using porous silicon carbide, a chemically inert semiconductor^7^. Applications of porous gallium nitride and related heterostructures already appear in various areas, among which are the high sensitivity hydrogen gas sensors^8^, water splitting^9^, energy storage^10,11^, development of a new substrate transfer technology^12^, fabrication of light-emitting devices^13,14^ and microcavities^15^.

Up to now, however, there is no full understanding of specific electrodynamics inherent for the porous thin films, which distinguish them from the continuous ones. In this regard, we must note that the probability of photon emission is controlled by the coupling of an optical transition with electric fields of both exciting and emitted photons. At an interface between two continuous media – air and semiconductor with dielectric constants ε_0_ and ε_1_, respectively – an electric field *E*_1_ inside the semiconductor is expressed in terms of the in vacuum electric field E_0_ as *E*_1_ = *E*_0*_ε_0_/ε_1_ in line with the well-known boundary conditions^16^. As a result, the field *E*_0_ in air is several times higher than in the semiconductor. On the other side, the porous medium can be considered as an ensemble of dielectric spheroids, whose inherent feature is the effect of depolarization^17^. Therefore, the electric field inside a spheroid differs from what it is in a continuous medium, and it is determined by the so-called “depolarization factor” which depends on the aspect ratio and on the orientation of this spheroid with respect to the external electric field^16,17^. Substantial enhancement of local field is also possible at the void edging, in surrounded material^18^. Such a “dielectric” enhancement taking place in semiconductor nanoparticles and near voids is not as high as plasmonic-induced enhancement in metallic particles, but it could explain the order of magnitude of PL enhancement in the porous materials.

It is worth noting that the porous medium can exhibit another unique effect, such as a strong optical anisotropy (PL polarization), which was observed in the porous Si and explained assuming constituent nanoparticles to have spheroidal shapes^19^. Recently, dielectric metasurfaces based on silicon nanopillars were suggested to work as a dielectric mirror and a band filter^20^. Note that the metasurfaces, whose theory and implementation are rapidly developing now, present a novel way to control light direction and polarization^21^. The random grating arising due to structural inhomogeneity in a surface layer can also control the optical properties^22^. Porous material with their specific light polarization and enhancement can be used as such random metasurfaces.

A photonic effect of paramount significance in elongated dielectric nanoparticles is the directional output of emitting light (dielectric “nano-antenna”) that allows one to enhance the light extraction efficiency. The nanowire antenna enable the implementation of stable, single-photon emitters^23,24^, and permit to create all-dielectric light concentrator with subwavelength volume^25^. One can notice a certain link between this effect and the so-called “lightning rod effect” in sharp dielectric structures, which recently attracted much attention^26,27^. Thus, the nanoparticles forming the porous material can enhance its light extraction efficiency. We highlight that analysis of a porous system, which can exhibit plenty of electrodynamic and photonic effects, is not a simple task. For GaN and other wide band gap porous materials, it can only be done properly by taking into account the excitonic nature of emission.

In the present work, we report on the detailed studies of excitonic emission in GaN films which experience post-growth porosification. We show that the emission related to free excitons dominates up to room temperature in our samples. The marked increase in the PL intensity from bulk to porous GaN films is mainly correlated with the enhancement of the local electric field and with the increased luminescence extraction ability, which both depend on the porosity level. In addition, we demonstrate that texturing of our samples reduces the efficiency of nonradiative recombination channels. To evidence these effects, absent in as-grown continuous GaN films, we exploit time-resolved PL (TRPL) spectroscopy in a wide temperature range.

## Experimental Results

**Figure 1:**
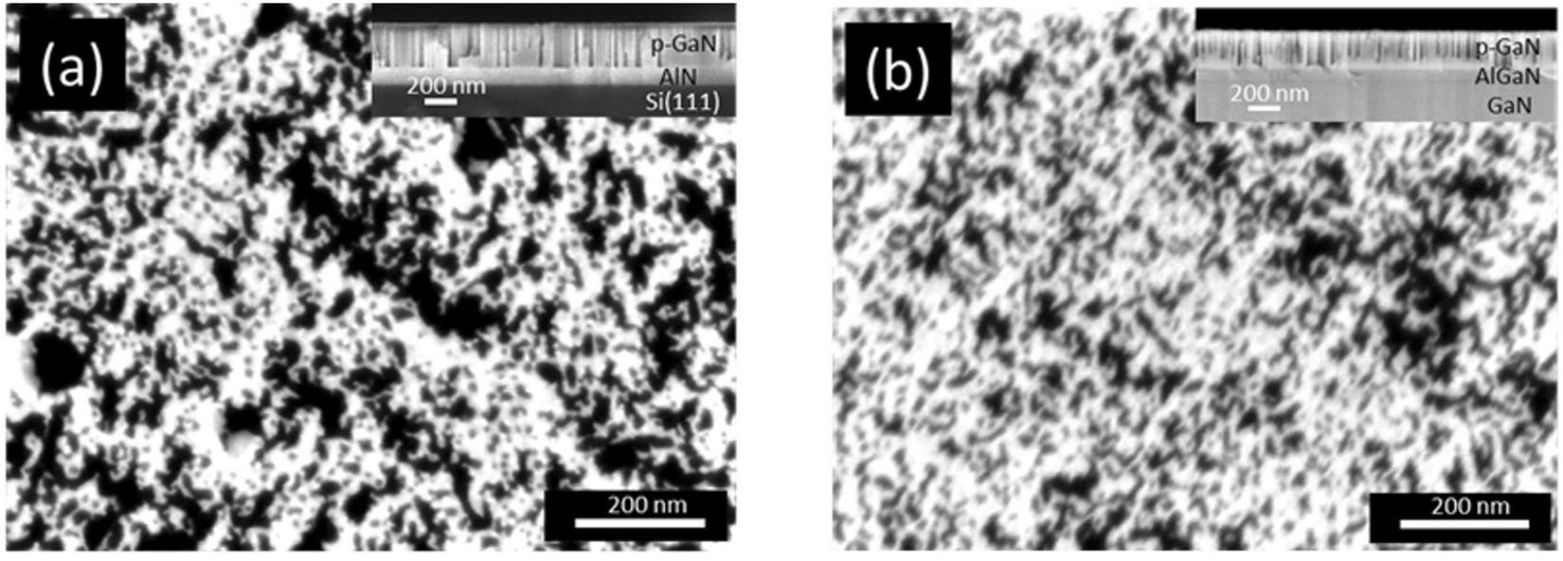
(**a**) Top-view scanning electron miscroscopy (SEM) image of porous GaN on AlN/Si(111). **(b)** Top-view SEM image of porous GaN on MOCVD-GaN template. Insets are cross-section view SEM images.

The experimental results were obtained using two samples: porous GaN on (111)Si and porous GaN on a MOCVD grown, (0001)-oriented GaN template, denoted hereafter as *p-GaN*/(111)Si and *p-GaN*/MOCVD-GaN, respectively. Details of their fabrication are given in Methods. Scanning electron microscopy images of the samples are given in Fig. 1. The pores in these samples occupy 48% and 27% of total surface, respectively. The characteristic diameters of the pores are in the range of 10–80 nm, while the remnant GaN dimension varies from 10 nm to more than 100 nm. Thanks to the pores, the surface area is increased by 20 times compared with original epilayers. Other details of structural characterization can be found in the Supplementary.

**Figure 2:**
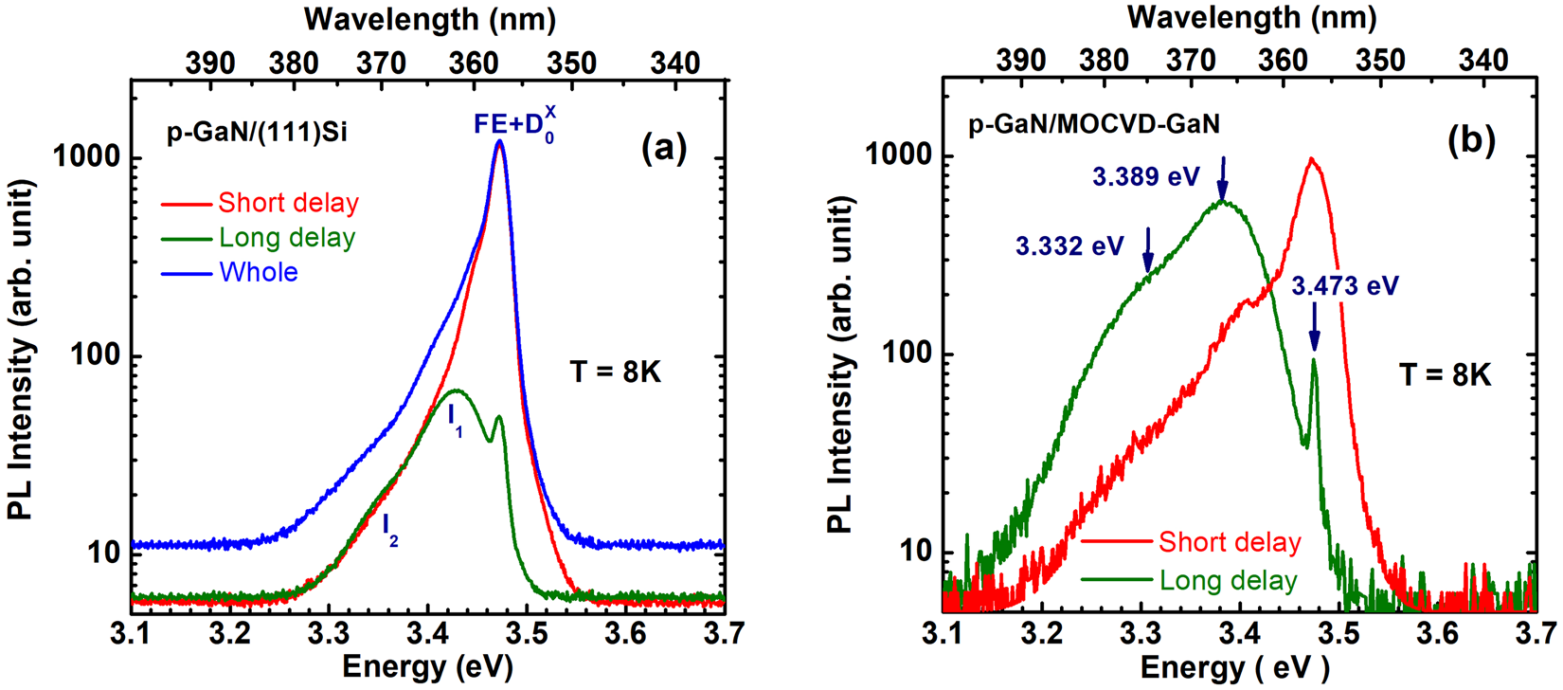
Time-integrations of time-resolved PL spectra collected at low temperature from the two porous GaN samples **(a**) p-GaN/(111)Si and **(b)** p-GaN/MOCVD-GaN measured using 300 grooves/mm grating. Restricting the integration to long delays after the laser excitation pulse permits is to clearly reveal the existence of stacking faults leading to I_1_ (3.42 eV) and I_2_ (3.35 eV) related recombinations. In the high energy range the PL is due to confined free (FE) and donor-bound (D_0_X) excitons recombination.

We begin the description of optical results by the presentation of overview PL spectra measured in a large enough spectral range in the *p-GaN*/(111)Si and *p-GaN*/MOCVD-GaN samples. Figure 2(a) shows a logarithmic representation of a PL spectrum of *p-GaN*/(111)Si obtained by integration over time of the TRPL spectra recorded using a streak camera (see Methods). Various conditions were selected for the time integration to evidence the composite nature of the transient PL spectra: (*i*) over the whole delay between two laser successive pulses, (*ii*) over a short range of time delays (0–0.3 ns), and (*iii*) over the long range of time delays (0.3–1.2 ns). A strong peak recorded at 3.473 eV in both samples corresponds to the radiative recombination of a mixed population of excitons bound to neutral residual donors (D_O_X) and free excitons (FE). Lower in energy, we detect in the *p-GaN*/(111)Si sample two broad bands: I_1_ at 3.42 eV and I_2_ at 3.35 eV, respectively (see Fig. 2(a)). They are evidently related to the stacking faults (see the Supplementary for details). Similar bands slightly shifted in energy are also observed in the *p-GaN/*MOCVD-GaN sample. Interestingly, in this sample of better quality but lower porosity, the contribution of these bands is enhanced as compared with the *p-GaN*/(111)Si. This finding impels us to conclude that the porosity prevents the transport of photo-excited carriers to such defects.

**Figure 3:**
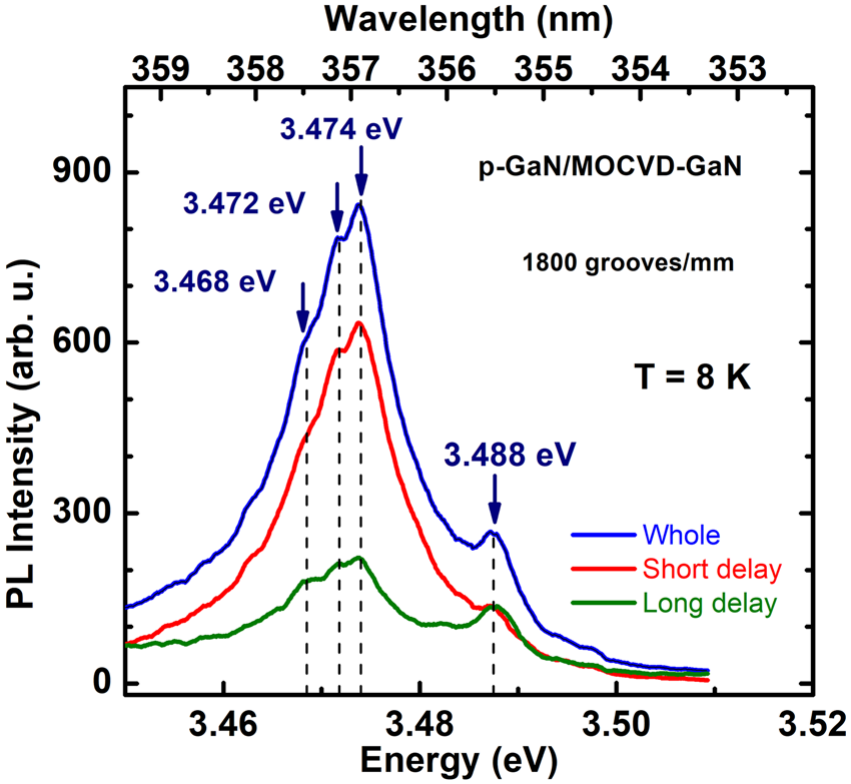
High resolution PL spectra of the porous GaN grown on the MOCVD-GaN template. Dashed lines are guides to the eyes. The 3.488-eV line originates from the MOCVD GaN template.

Under these measurement conditions (300 grooves/mm grating), the fine structure of the basic peak can hardly be resolved. However, using a 1800 grooves/mm grating in the study of *p-GaN/*MOCVD-GaN (Fig. 3) we were able to resolve the PL features at 3.474 eV, 3.472 eV and 3.468 eV, which can be, respectively, attributed to the FE, Si- and O-related D_O_X^28,29^. The 3.488 eV line here corresponds to the MOCVD template. We underline that these donor-related features were usually resolved in bulk structures of high enough qualities. Their resolution in the PL contour evidences a good quality of this sample, as well as the absence of noticeable contamination during the MBE growth. A slight distortion of the global shape is observed between spectra built by integration at short delay to long one, which indicates that decay times of bound excitons are longer than the decay times of free excitons at low temperature, as commonly known. It is worth noting that in bulk GaN the D_O_X contribution dominates in the low temperature range, whereas FE contribution dominates at temperatures higher than ~30–35 K^30^. Measurements of the spectra with temperature variation allow us to distinguish these components.

**Figure 4:**
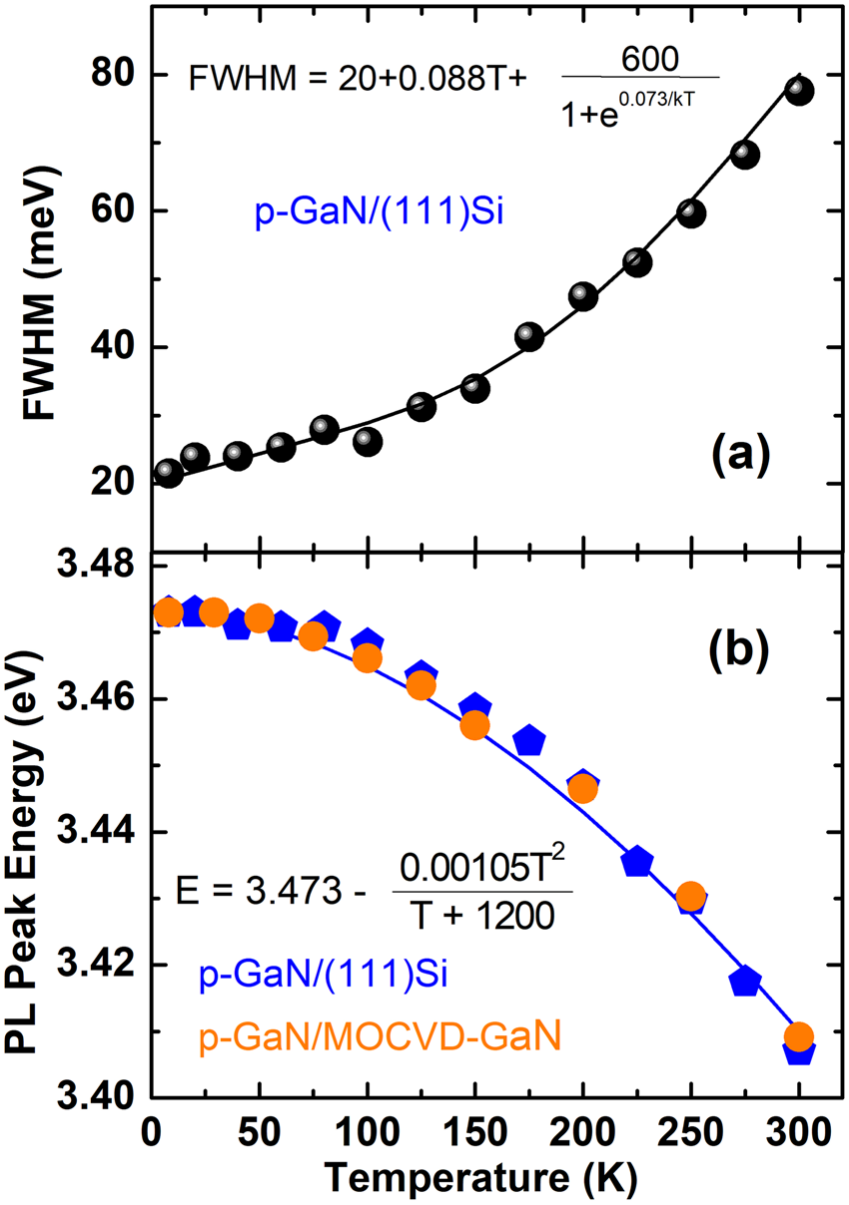
(**a**) Evolution of the full width at half maximum of the (FE + D_0_X)–related PL against temperature: black spheres correspond to the experiment data on p-GaN/(111)Si whilst full line is the result of the fitting. (**b**) Temperature-induced redshift of the (FE + D_0_X)-related PL: blue pentagons and orange circles denote the experimental data on p-GaN/(111)Si and p-GaN/MOCVD-GaN, respectively. The full line shows the fit using Varshni’s equation.

Previously performed investigations of the PL spectra of nanowires versus their average sizes demonstrated that a free exciton emission originates predominantly from those which have 12–19 nm in diameter (ref.^29^). These sizes match reasonably the sizes of GaN continuous regions in our porous films. The full widths at half maximum (FWHM) of the basic excitonic features in spectra are 20 meV at low temperature, as shown in Fig. 4(a) for *p-GaN*/(111)Si. The FWHM value gradually increases when increasing temperature, thanks to interactions of excitons with phonons. The excitons probe the whole phonon bath (acoustic and optical phonons) and the average phonon energy is a weighted contribution of all the phonon states through the whole Brillouin zone^31–33^. The fit of temperature dependence in Fig. 4(a) gives an average phonon energy of 73 meV. Figure 4(b) shows that the main PL peak redshift when the temperature increases. The absence of S-shape is an indication of negligible localization effects. This redshift can be fitted using a Varshni’s law^34^ with parameters which are consistent with previously reported ones. Note that no significant difference has been observed between the temperature dependencies measured in two studied samples: *p-GaN*/(111)Si and *p-GaN*/MOCVD-GaN.

**Figure 5:**
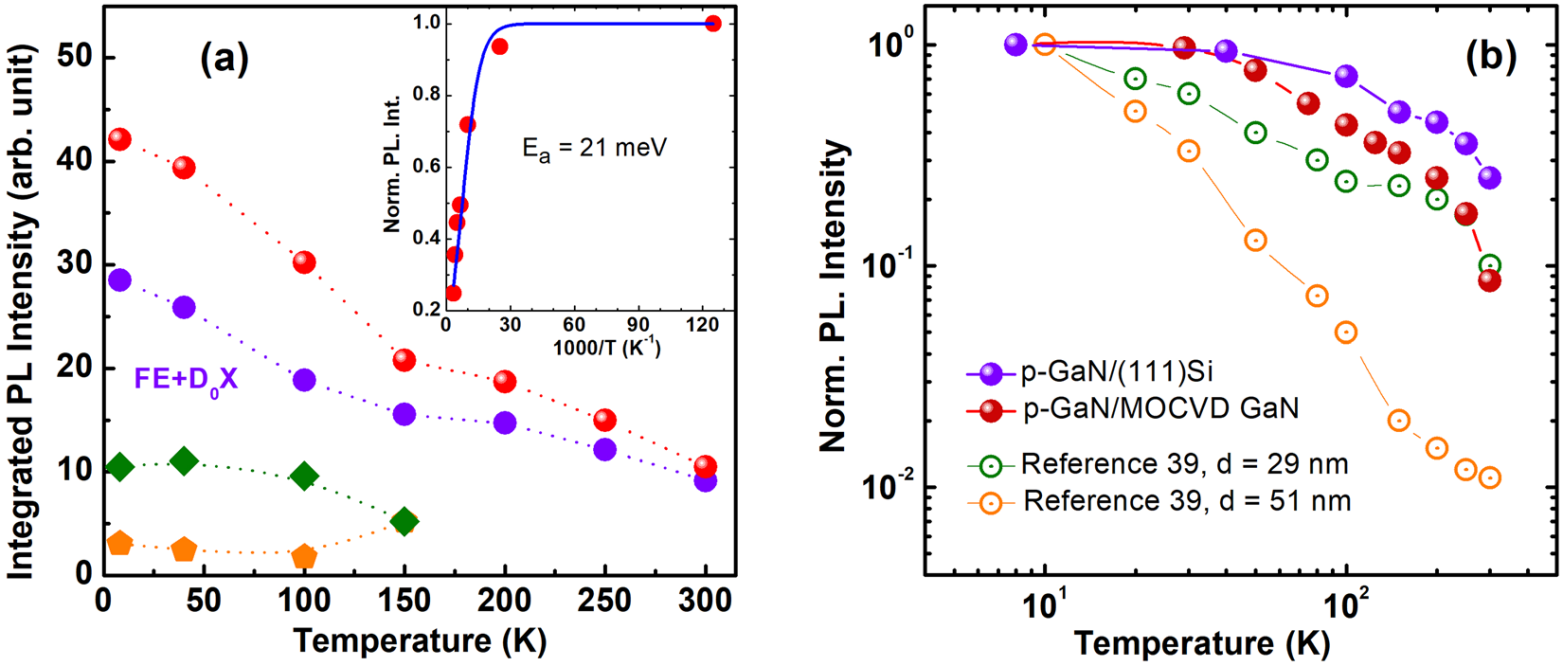
Evolution of the PL intensity against temperature measured in different samples. (**a**) Integrated PL intensity of different bands in p-GaN/(111)Si sample: red spheres correspond to the total spectral integration, purple dots, green diamonds and orange pentagons correspond to (FE + D_0_X), I_1_ and I_2_ related transitions, respectively. Note the thermal equilibrium reached after 150 K. Inset: Arrhenius plot of the PL intensity, which corresponds to an activation energy about 21 meV that is close to the free exciton binding energy of bulk GaN. (**b**) Normalized PL intensity for our two porous GaN samples in comparison with the results reported by Zettler *et al*. for GaN nanowires with different diameters^39^. Dotted and solid lines are guides to the eyes.

As it was previously reported^35^, the PL intensity is very strong in case of GaN material prepared in terms of a nanorod-like texturing. In our *p-GaN*/(111)Si sample, the intensity quenches by a factor ~4 between low and room temperature, as indicated in Fig. 5(a). The inserted Arrhenius plot of the intensity corresponds to an activation energy of 21 meV that is close to the free exciton binding energy of bulk GaN. This quenching of intensity is obviously small as compared with other reported data. In an undoped GaN continuous film, the intensity decreases by two orders of magnitude^36^, and a GaN epilayer on a Si(111) substrate exhibits a PL quenching by a factor of ~20^37^.

The weak intensity variation from low to room temperature in *p-GaN* evidences the suppression of non-radiative recombination channels (migration of carriers towards defects) which was suggested previously^38^. However, the intensity of the defect-related emission bands is weak (see Fig. 5(a)). The I_1_ and I_2_ lines collapse when increasing T and cannot be distinguished for the temperatures higher than 150°K. This corresponds to what is generally expected from the published data^35,39–42^. Thus, the redistribution of total excitation in their favor or disservice could not significantly change the balance. Therefore, we assume that the principal reason of the PL robustness has to be an enhanced recombination rate of excitonic emission which renders uncompetitive the slow recombination rate at these defects.

In Fig. 5(b), we present the evolution of the normalized PL intensity against temperature for our two *p-GaN* samples in comparison with the results reported by Zettler *et al*. for GaN nanowires with different diameters^39^. We clearly observe that the robustness of the *p-GaN* PL intensity with temperature is similar to what is reported in ref.^39^ for narrow wires with diameters smaller than 30 nm. When considering our two *p-GaN* samples, we remark that the higher porosity of the *p-GaN*/(111)Si sample leads to the most robust PL intensity with temperature (even if it contains many defects).

**Figure 6:**
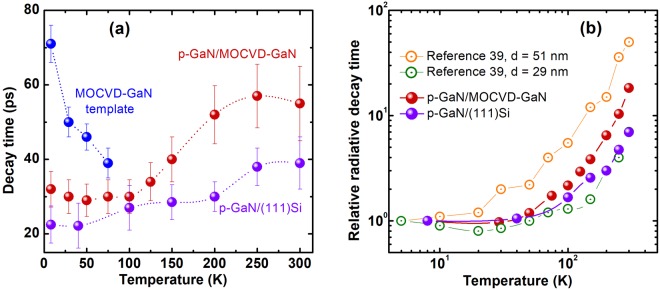
(**a**) Evolution of the characteristic decay times of basic emission bands in p-GaN/(111)Si and p-GaN/MOCVD-GaN with temperature. (**b**) The relative radiative decay times (renormalized to their low temperature values) of our porous GaN in comparison with the results reported by Zettler *et al*. for GaN nanowires with different diameters^39^, as a function of temperature. Dash and solid lines are guides to the eyes. The notations of marks are given in the plots.

We measure the decay times at different energies in the porous GaN to probe the decay kinetics of the FE and defect-related lines (see Fig. S4 in the Supplementary for the decay curves and particular data). The long decay times of both I_2_ and I_1_ bands and their decrease with the temperature rise are typical for the stacking faults related emission in III-nitride structures^43^. On the contrary, the decay times of FE lines in both *p-GaN* sample exhibit some increase with temperature, hence, a decrease of the recombination rate (Fig. 6(a)). This effect results from the temperature-induced redistribution of the exciton k-vectors through the whole k-space. Note that the recombination rate is proportional to the exciton population with wave vectors within the light cone, that is to say near k = 0. Such “bottleneck” situation leads to a bit delayed recombination times with thermally-induced exciton vector dispersion.

We remark that the decay time values of the 3.473 eV line at low temperature (22 ps in *p-GaN*/(111)Si and 32 ps in *p-GaN/*MOCVD-GaN) are fully consistent with what is generally measured in nanowires of sizes ranging from about 10 nm to 80 nm^39,42,44–47^. Also interesting are the comparative behaviors of the PL from the MOCVD GaN template and *p-GaN* in the same sample (Fig. 6(a)). When increasing temperature, the decay time of the PL of the GaN template decreases rapidly (similar to the quenching of the PL intensity) as a manifestation of efficient non-radiative recombination channel(s), while the decay times of porous p-GaN are not so much affected and even tend to increase with the temperature rise, due to the above-discussed effect of exciton k-space filling.

Such robustness of the PL of *p-GaN* invites us to enlarge the comparison with more many samples, in particular, with the ensembles of nanowires. In Fig. 6(b) are plotted the temperature evolution of the PL decay times renormalized to their low-temperature values for our two *p-GaN* samples in comparison with the results reported by Zettler *et al*. for GaN nanowires with different diameters^39^. We clearly observe that the decay of the *p-GaN* PL with temperature is similar to what is observed in ref.^39^ for narrow wires with diameters ~30 nm. The corresponding non-radiative recombination times are constant in the high temperature range^39,48^. Although the morphologies of our samples are really different from those of ref.^39^, we observe similar T^3/2^ dependence increasing when the temperature T rises.

## Analysis and Discussion

Our principal optical findings in porous GaN can be summarized as revealing excitonic recombinations with high recombination rates that are dominating up to room temperature with a very weak decrease in intensity. This is accompanied by the suppression of non-radiative channels and effective light extraction from the texturized porous surface.

To analyze these effects the constituent GaN nanoparticles can be considered in a first approximation as spheroids with a frequency-dependent complex dielectric functions ε_1_(ω) surrounded by air with dielectric constant ε_0_ = 1. This is a limiting case for high porosity. The size estimations, performed during the sample characterization, allow us to regard them as a representative prolate spheroid, whose long axis *c* coincides with the rotation axis, while axes *a* = *b*. The applied field *E*_0_ induces a polarization *P* of the ellipsoid, which modifies the field inside the spheroid, *E*_1_, and changes the potential outside of it. The boundary conditions dictate the continuity of the tangential component of the electric field vector on the spheroid surface and a rapid dropping the electric field *E*_2_ away from the ellipsoid.

The uniform internal field inside the spheroid *E*_1_ is determined by the relation^16,19^

1 $${E}_{1}^{i}={(1+{L}^{i}(\beta -1))}^{-1}{E}_{0}^{i}.$$

Here, *β* = ε_1_(ω)/ε_0_, where the dielectric constant ε_1_ in the vicinity of excitonic resonances is taken equal 8^49^. *L*^*i*^ is the depolarization factor along *i*-th axis, expressed via elliptic integrals. Its value does not depend on the size of the spheroid, but only on its aspect ratio^17^. The calculation of depolarization factor dependences with the *a/c* ratio gives *L* values ~0.02 for *E*_0_ directed along the longest axis *c* and about 0.5 when *E*_0_ is parallel to the short axes *a* = *b*. These factors correspond to values *E*_1_ = 0.9*E*_0_ and *E*_1_ = 0.2*E*_0_, respectively. The later value matches conventional experimental conditions, when laser beam impinges perpendicular to the growth surface, that is to say when the Poynting vector of the photon in the laser beam is parallel to the normal of the semiconductor surface. Note that even a small angle between the spheroid main axis and the laser beam provides the deviation of the *E*_1_ value in favor of its enhancement. Also, through the ensemble of the nano-particles, some can be found which have the shape of the oblate spheroids; for them *E*_1_ ≈ *E*_0_. For the pores, one should substitute the GaN dielectric constant and other parameters by those of air, i.e. *β* = ε_0_/ε_1_(ω). The estimation shows that it is possible even to obtain a weak resonance with the enhancement of the input electric field by an order of 1.5–2 in the vicinity of a pore. In any case, all these values are higher than the expected *E*_1_ = 0.12*E*_0_ for the planar GaN/air interface. Thus, PL intensity can be enhanced by several times in the porous GaN films as it has been indeed observed in experiments. In porous InGaN/GaN quantum well structures, this depolarization effect can also contribute to the weakening the quantum confined Stark effect, which was observed experimentally in ref.^38^.

Importantly, in spite of many stacking faults in the *p-GaN*/(111)Si sample, the spectrally  integrated intensity of its excitonic PL band is comparable with that in the better quality *p-GaN/*MOCVD-GaN sample. This evidences an enhancement that may be just due to the concentration electromagnetic field at the numerous nanoparticles and pores. Also, the decay time in this *p-GaN/*(111)Si sample is the smallest among presented, i.e. the recombination rate is highest. In the *p-GaN/*MOCVD sample with a twice smaller porosity, the recombination rate is decreased by the factor 2, as compared with the *p-GaN/*Si one. The combination of these two features – enhanced intensity with high recombination rate – is characteristic of the Purcell effect^50^. We assume that this effect is controlled by the porosity in our samples.

Concerning the role of the inherent defects, we should underline that in the porous material the defect-related PL intensity is by order of magnitude less in average than the intensity of a basic line. With increasing porosity, the intensity of the defect PL can drop noticeably due to the suppression of carrier transport to these defects. However, we assume that the main reason is the promotion of the recombination of free excitons because of the higher recombination rate enhanced by the Purcell effect.

Thus, two consequences of the Purcell effect have crucial significances. First, the enhanced recombination rate in the porous material, which as itself promotes the intense emission. Second, the fast recombination in *p-GaN* makes impossible the excitation transfer towards the inherent defects characterized by slow recombination. The comparison of PL intensity with the temperature rise in the porous and continuous films is the best illustration of these phenomena. We highlight that the similarity between different textured systems, fabricated by different ways evidences the generality of analyzed optical processes. It is a strong argument pleading in favor of the validity of the proposed electrodynamic approach.

## Conclusions

We investigated porous p-GaN thin films grown by MBE on a Si(111) substrate and on an MOCVD GaN template, which display different porosity: the total area occupied by pores was 48% and 27%, respectively. The surface of the air/semiconductor interface in the porous material is higher by a factor of 20 than that of a planar surface. An enhancement of PL intensity and its stability with temperature were observed in correlation with the porosity. We defined a dominant factor which produces this enhancement as the depolarization effect at nanoparticles and pores resulting in the increase of the local electric fields. We found that the recombination rate (inversely proportional to the characteristic PL decay time) is proportional to the porosity degree and, hence, to the electric field concentration in these samples. The obtained data are suggestive of the Purcell effect that promotes the exciton-photon coupling via local enhancement of electric field in a small volume. The dielectric antenna formed by the nanostructures comprising the textured surface and the enhanced interface area promote the efficient light extraction from the porous samples. We did not observe any evidence of strong non-radiative recombination and we consider it as the signature of suppressed carrier transport towards defective regions. Excitonic features turned off to be well pronounced up to room temperature and transient behavior of PL corresponds to those typical of a semiconductor with strong excitonic resonances. We believe that our findings can be useful for further development of porous wide-gap materials for nanophotonic applications. These profoundly two-dimensional materials can be considered as a prototype platform for excitonics, with specific properties controlled by the surface texture.

## Methods

The samples were fabricated by molecular beam epitaxy (MBE) using solid-sources for Al, Ga and Si elements and NH_3_ for N. They were grown on a Si(111) substrate and on an MOCVD-grown GaN template deposited on sapphire. The sample grown on Si(111) substrate was constituted by a 100 nm-thick AlN buffer layer followed by a 270 nm-thick GaN layer transformed to the porous material. The sample grown on a sapphire (0001) substrate comprises a MOCVD-grown 2.5 µm-thick GaN layer (template) followed by an Al_0.2_Ga_0.8_N (20 nm) etch-stop layer and the 245 nm-thick GaN layer, also grown by MBE and subjected to porosification. The porosity could be affected by different crystalline quality and doping concentration. To diminish the influence of these factors the top GaN layers in both samples were grown by the same MBE technique using the same growth conditions.

For porosification, a self-organized Si_x_N_y_ nanomask was formed by exposing the GaN surface to a Si flux. The samples were then heated at 900 °C under vacuum in order to sublimate the GaN left uncovered by the Si_x_N_y_ nanomask. To prove the stability of the non-polar samples with porosification, we studied samples with thicker GaN layers (2-3 µm) prepared with an *ex situ* Si_x_N_y_ mask. In this case, we can evaluate the anisotropy of the evaporation rate. We found that the evaporation rate of the vertical non-polar planes is 30–40 times smaller than the horizontal planes. This is indicative of the high thermal stability of these planes.

In both samples, the same procedure is applied to fabricate the porous GaN, i.e. the same Si exposure time, sublimation time, and temperature. Nevertheless, we observe almost twice larger porosity (0.48) in the sample on Si(111) than on sapphire (0.27), likely because of the sublimation rate is enhanced near the numerous dislocations.

The pores density and their characteristic sizes were characterized by image processing software that indicating the developed surface in the porous films is higher by an amount of 20 than the planar surface. This crude calculation permits to frame an enhancement of the light extraction efficiency. These data were also used to determine the values of average dielectric permittivity, as described in the Supplementary. We underline that the extracted value characterizes a porous system as a whole that may be useful, e.g., in the analysis of its response as a metasurface.

Other details regarding the realization and characterization of our samples can be found in preceding papers of some of us, including data on temperature dependent CW spectroscopy^38,51^. In particular, the PL intensity of the porous GaN was comparable to that in the highest quality reference sample of bulk GaN.

The description of the used TRPL set-up can be found in ref.^52^. The samples are excited using a 266-nm laser line, a third harmonic generation of a mode-locked Ti:sapphire laser. The repetition rate of the laser is set at 82 MHz, in order to sweep recombination times of the samples in short time ranges. The samples were cooled down in a helium follow cryostat with temperature varied from 8 K to 300 K for temperature-dependent investigation of the recombination times. The emitted light from samples was recorded by a 500IS Hamamatsu spectrometer and then temporally resolved by a Hamamatsu-C10910 streak camera, whose time-resolution is about 3 ps. The grating of the spectrometer is selected between either 300 grooves/mm or 1800 grooves/mm for different spectral resolutions at the TRPL measurements. During TRPL experiments, we maintain the same excitation power for both samples. Its average value measured before the cryostat window was P_0_ = 1 mW that corresponds approximately to the excitation power density P_0_/S ~10 W/cm^2^ when a laser beam is focused in a spot with a radius of ~0.05 mm.

## Electronic supplementary material

Supplementary information
