# Supplementary material for: Enhanced excitonic emission efficiency in porous GaN
Source: Sci Rep. 2018 Oct 25;8:15767. doi: 10.1038/s41598-018-34185-1 (PMC6202416; doi:10.1038/s41598-018-34185-1)
Supplement: Supplementary file 1 — Supplementary information [file 41598_2018_34185_MOESM1_ESM.docx]

**Supplementary information for**

**“Enhanced excitonic emission efficiency in porous GaN”**

Thi Huong Ngo^1,2,*^, Bernard Gil^1,3,+^, Tatiana V. Shubina^3$^, Benjamin Damilano^4^,

Stéphane Vezian^4^, Pierre Valvin^1^ and Jean Massies^4^

*1: Laboratoire Charles Coulomb, CNRS and Université de Montpellier, CC074, 34095 Montpellier Cedex 5, France*

*2: Faculty of Science and Technology, Meijo University, 1-501 Shiogamaguchi, Tenpaku-ku, Nagoya 468-8502, Japan*

*3: Ioffe Institute, 194021 St Petersburg, Russia*

*4: Centre de Recherche sur l’Hetero-Epitaxie et ses Applications- CNRS- Rue Bernard Gregory, Sophia Antipolis, 06560 Valbonne, France*

*^*^*[*ngo@meijo-u.ac.jp*](mailto:ngo@meijo-u.ac.jp)

[*^+^Bernard.gil@umontpellier.fr*](mailto:+Bernard.gil@umontpellier.fr)

*^$^Shubina@beam.ioffe.ru*

1. **Samples characterization**

Results presented here were obtained on 250-nm thick two-dimensional layers of GaN grown by Molecular Beam Epitaxy (MBE) on Si(111) covered by a 100-nm-thick AlN layer and on a GaN template (2.5 µm-thick GaN layer followed by a 20-nm-thick Al_0.2_Ga_0.8_N layer) fabricated by Metal-Organic Chemical Vapour Deposition (MOVCD). Structure of the samples are represented in figure S1(a). The lower part is not porous as shown on the SEM image of GaN grown by MOCVD on sapphire substrate (figure S1(b)). The 2D-GaN is compressively stressed while the porous GaN is almost relaxed. Therefore, the PL spectra are well distinguished (see figure S2). Note that the contribution of the PL from the template is significantly less than the PL from the porous GaN due to the small length of excitation propagation into the wide-gap material (~100 nm).

| (a)  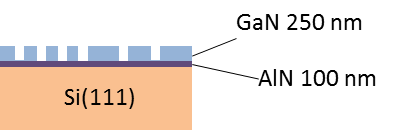  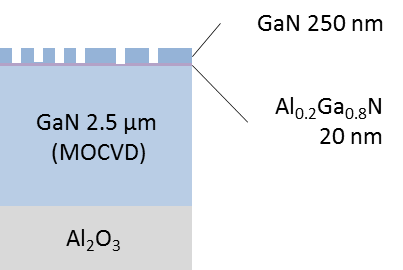 | | (b)   |
| --- | --- | --- |
| ***Figure S1: (a)*** *Structures of porous GaN on Si(111) and of the porous GaN on MOCVD-GaN on sapphire (0001) substrate.* ***(b)*** *Cross-section of scanning electron microscopy of the porous GaN/**MOCVD-GaN sample.* | | |

The dislocation densities in these samples were, respectively, 3x10^10^ cm^-2^ and 5x10^8^ cm^-2^. The samples were made porous using the selective area sublimation (SAS) technique^i,ii^. We assumed that the different dislocation densities in the samples grown on different buffers should influence the porosity degree. Indeed, the pores occupied 48% and 27% of total surface, respectively.

Scanning electron microscopy studies (see, e.g., figure 1 of the main text) exhibited that in the *p-GaN*/Si(111) the pores are of nanometric size (typically with diameters in the range of 10-80 nm) in the direction perpendicular to the growth plane and with a depth of 250 nm. The remaining GaN can be seen as a network of interconnected nanorods (nanowires) as revealed by the cross-section SEM images. The lateral dimensions of continuous GaN can vary on a broad range from less than 10 nm to more than 100 nm. We believe that sublimation is so directional and forms the nanorods most probably in relation to the fact that non-polar planes are the most stable planes (cleavage planes) for wurtzite nitride semiconductors. This conclusion was confirmed by technological experiments, described in the Methods. In the *p-GaN* grown on the MOCVD template, where the pores occupy about quarter of the total area, more extended GaN net regions were observed.

The pores density was characterized by an image processing software that specifies about 260 (resp. 350) pores occupy a surface of 0.48 µm^2^ (resp. 0.27 µm^2^) for porous GaN on silicon (resp. MOCVD-GaN) of 1x1 µm^2^ surface of the samples. Browsing all holes, the software gives their average perimeter. The values of the total perimeters at the air/semiconductor interface are 73 µm (resp. 76 µm) leading to a total air/GaN lateral surface of 18.25 µm^2^ (resp. 19 µm^2^). Further adding the 0.48 µm^2^ (resp. 0.27 µm^2^) one obtains the crude values of 18.73 µm^2^ (resp. 19.27 µm^2^) by an amount of about 20 just by the porosification of the GaN.

Although the surface morphology is more likely random or fractal^iii^ (see figure 1), we can assume these pores to be round in plane. We model the amount of GaN corresponding to about 52% (resp. 73%) of the total surface for *p-GaN* on Si (resp. on MOCVD-GaN) in terms of nanorods of GaN of an identical size. Such rough estimation allows us to obtain a characteristic nanorod diameter of 12 nm (resp.19 nm) for p-GaN on silicon (resp. GaN). The conditions to face a dielectric confinement, that can be found in the work of L. Keldysh^iv^, are not fulfilled in our samples, the effective diameters of our rods (about one order of magnitude larger in terms of size than the excitonic Bohr radius in GaN) being too large for that. Therefore, we believe that the optical properties of these samples will be more affected by the texture of the GaN than by any confinement of the excitons. This is well consistent with experimental data obtained by TRPL.

1. **Determination of average dielectric permittivity**

The important characteristic is an averaged dielectric constant which characterises the porous GaN as an effective medium. This parameter can be useful in consideration of a porous two-dimensional layer as a metasurface. Computing the dielectric constant of such composite GaN-Air material is tricky^v,vi^. We find the values of the average dielectric permittivity <ε> of *p-GaN* samples using Clausius-Mossoti equation^vii^ as

$$<\varepsilon> = \frac{(1+2p)\varepsilon_{0}+ 2\left( 1-p \right)\varepsilon_{1}}{(1-p)\varepsilon_{0}+ \left( 2+p \right)\varepsilon_{1}} \varepsilon_{1}$$

Taking again $\varepsilon_{1}=8$ for GaN and for the porosity p = 0.48 or 0.27, this calculation gives <ɛ> = 4.0 for our *p-GaN* on Si and <ɛ> = 5.6 for the *p-GaN* on MOCVD-GaN, which indicates both a substantial reduction of the average dielectric constant and its dependence on the porosity. These reductions may impact the exciton oscillator strength, that is the value related to the radiative decay time, making the TRPL a suitable investigation tool.

**III. Optical characterization**

The contribution of both the porous GaN and 2D-GaN grown by MOCVD can be observed in the PL spectra.


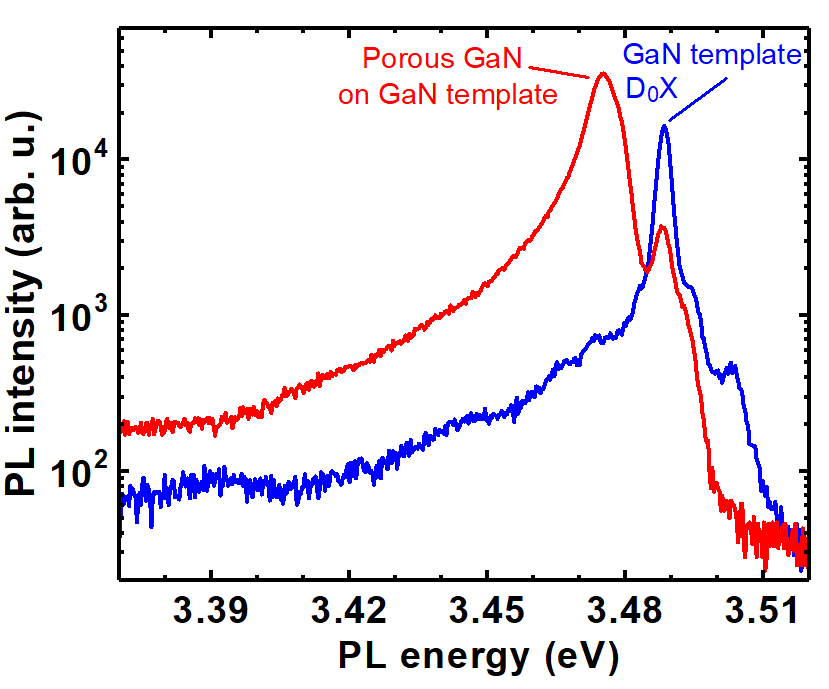


***Figure S2:*** *Photoluminescence spectra of the p-GaN and the MOCVD-GaN template.*

The typical temperature variation of PL spectra from porous GaN on the MOCVD GaN is presented in figure S3. It reproduces in general the variation in perfect bulk GaN. The temperature dependencies of characteristic decay times of combined emission bands are given in figure S4(a) for *p-GaN*/(111)Si. Evolution of the decay times of particular bands in this sample is shown in figure S4(b).


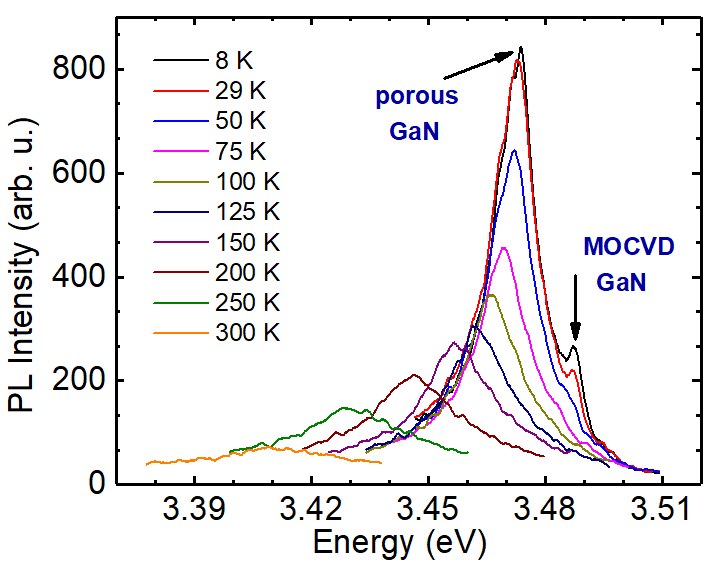


***Figure S3:*** *Temperature dependent PL spectra measured in the p-GaN/MOCVD-GaN.*

| 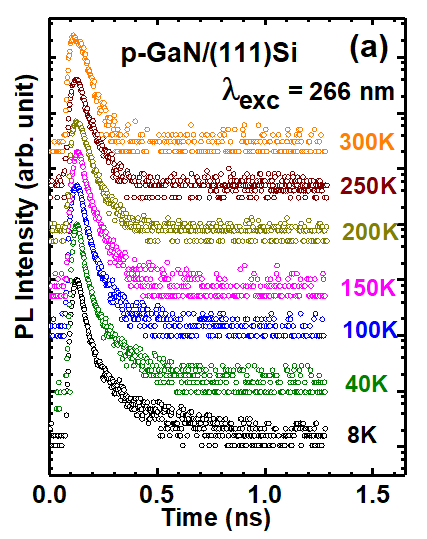 | 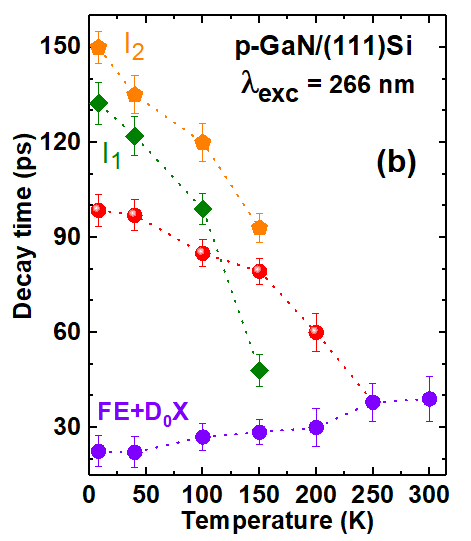 |
| --- | --- |
| ***Figure S4: (a)*** *Typical PL transients (combined contributions of FE+D_0_X, I_1_ and I_2_) recorded at different temperatures in the p-GaN/(111)Si sample.* ***(b)*** *Evolution of the decay times: purple dots, green diamonds and orange pentagons for (FE+D_0_X), I_1_ and I_2_ related recombinations, respectively. Red spheres represent the average values obtained by approximated fitting the transients with single characteristic decay exponent. Dotted lines are guides to the eyes.* | |

Our studies exhibited the emission bands related to the presence of stacking faults in our porous GaN samples. Detailed investigations of the origin of these lines were previously published, which include correlations between PL and cathodoluminescence studies. An excellent article reviewed the origin of these recombination lines^viii^. Both of them correspond to stacking faults of the ABABABAB wurtzite stacking, namely I_1_ at 3.42 eV (ABABCBCBC… stacking) and I_2_ (ABABCACAC… stacking) at 3.35 eV. We remark the lack of extrinsic E-type stacking faults in our samples (ABABCABAB… stacking).

Performed TRPL measurements show that the rate of emission originating from the stacking faults is obviously lower than the recombination rate of the higher energy feature at 3.473 eV, as these defect-related bands are the most clearly seen in spectra integrated over the long range of time following the excitation pulse. Currently, the longer low-temperature decay times of emission bands related to the staking faults is attributed with consensus to the electric field effects at the interfaces between the stacking fault region and the host GaN crystal.

The characteristic PL decay curves measured in porous GaN can be found in figure S4(a). They were obtained by an analysis done in general, neglecting that the decay time varies with both the energy and temperature and that the distortion of PL towards low energies takes place at long delays. These curves clearly demonstrate that the basic decay is fast and a weak longer-decaying tail disappears with the temperature rise. Figure S4(b) shows the temperature evolutions of the PL decay times, extracted from the exponential fit of the TRPL spectra. By carefully examining the decay of the PL lines, we can derive selective decay time values of I_1_ and I_2_ up to 150-200 K.

The PL spectra were stable during all TRPL measurements in a wide temperature range. Such a situation is fully different from what was observed on porous Si, where ageing under ambient conditions resulted in PL energy shifts due to the confinement effect^ix^ and surface contamination effect by oxygen or OH radicals^x^. Owing to the chemical inertness of GaN, such PL modification with ageing was not observed under similar experimental conditions.

**References**

i) Damilano, B., Vezian, S. & Massies, J. Photoluminescence properties of porous GaN
and (Ga,In)N/GaN single quantum well made by selective area sublimation. *Opt. Express* **25**, 33243 (2017).

ii) Damilano, B., Vezian, S. & Massies, J. Mesoporous GaN Made by Selective Area Sublimation
for Efficient Light Emission on Si Substrate. *Phys. Status Solidi (B)* 1700392 (2017).

iii) Yogi, P. *et al.* [Porous Silicon's fractal nature revisited](https://www.sciencedirect.com/science/article/pii/S0749603618310280). *Superlattices Microstruct.* **120**, 141 (2018).

iv) Keldysh, L.V. Excitons in Semiconductor-Dielectric Nanostructures. *Phys. Status Solidi a* **164**, 3, (1997)

v) Bergman, D. The dielectric constant of a composite material – A problem in classical physics. *Phys. Rep.* **43**, 377 (1978)

vi) Milton, Graeme W. of referencing in *The Theory of Composites* (Cambridge UK, 2004).

vii) Cabuz, A. of referencing in *Metamaterials Modeling and Design* (ed. Felbacq, D. & Bouchitté, G.) (Pan Stanford, 2017).

viii) Lahnemann, J. *et al.* Luminescence associated with stacking faults in GaN. *J. Phys. D. Appl. Phys.* **47**, 423001 (2014).

ix) Voos, M., Uzan, Ph., Delalande, C., Bastard, G. & Halimaoui, A. Visible photoluminescence from porous silicon: A quantum confinement effect mainly due to holes? *Appl. Phys. Lett*. **61**, 1213 (1992).

x) Wolkin, M.V., Jorne, J., Allan, G. & Delerue, C. Electronic States and Luminescence in Porous Silicon Quantum Dots: The Role of Oxygen, *Phys. Rev. Lett*. **82**, 197 (1999).
